# Social Media Constructions of Alcohol as Emotional Coping and Social Connection in Ghana: Qualitative Study

**DOI:** 10.2196/102546

**Published:** 2026-09-16

**Authors:** Rachel GA Thompson, Evershed Kwasi Amuzu, Tricia Thompson, Mark Kwaku Gyasi, Orlando Harris, Glenn-Milo Santos, Jerry John Ouner

**Affiliations:** 1 Language Centre College of Humanities University of Ghana Accra, Greater Accra Ghana; 2 Institute for Global Health Sciences University of California, San Francisco San Francisco, CA United States; 3 Africa Interdisciplinary Research Institute Accra, Greater Accra Ghana; 4 Department of Linguistics College of Humanities University of Ghana Accra, Greater Accra Ghana; 5 Push Aid Africa Accra, Greater Accra Ghana; 6 Department of Family Health Care Nursing School of Nursing University of California, San Francisco San Francisco, CA United States; 7 Department of Community Health Systems School of Nursing University of California, San Francisco San Francisco, CA United States

**Keywords:** alcohol, social media discourse, emotional coping, social drinking, digital health communication, Ghana

## Abstract

**Background:**

Alcohol is a leading contributor to disease and injury worldwide; yet, it remains embedded in cultural practices associated with pleasure, social connection, and emotional relief. While global research has documented both the epidemiological burden and the social meanings of drinking, little is known about how alcohol-related meanings are circulated and normalized within African social media environments.

**Objective:**

This study explores how alcohol is represented in Ghanaian social media discourse, with particular attention to emotional coping, social interaction, and everyday well-being.

**Methods:**

The study draws on a qualitative dataset of publicly available user-generated content (N=189) from widely used social media platforms, Facebook and TikTok. It uses discourse analysis within a social constructionist framework, thereby treating language as a site where alcohol-related meanings are produced, negotiated, and circulated on online platforms.

**Results:**

Alcohol was routinely constructed as a resource for coping with stress, heartbreak, and economic strain, while also functioning as a facilitator of friendship, leisure, and communal belonging. Alcohol was further normalized as part of everyday mood regulation, with users presenting drinking as a means of enhancing relaxation, pleasure, and bodily comfort. A broader commercial-lay hybrid discourse was identified, in which promotional messaging, therapeutic claims, and peer communication became increasingly intertwined. Through these discourses, alcohol was associated with vitality, emotional restoration, and well-being.

**Conclusions:**

By situating these constructions within Ghana’s broader cultural and digital context, the study demonstrates how global narratives of drinking for self-care and leisure are adapted and recirculated through local social media practices. The study contributes one of the first qualitative analyses of alcohol-related social media discourse in West Africa and highlights the importance of public health communication strategies that engage with culturally meaningful and digitally circulating narratives rather than relying solely on risk-based messaging.

## Introduction

### Background

Alcohol maintains a paradoxical role within both public health and daily social life. On the one hand, it is a leading contributor to disease, injury, and mortality worldwide [1]. On the other hand, it remains deeply embedded in cultural practices associated with pleasure, social connection, leisure, and emotional relief [2,3]. In many societies, drinking is represented as a normal way to manage stress, strengthen relationships, and transition into relaxation or celebration. These meanings are not fixed but are continually produced, negotiated, and reinforced through everyday communication [4]. The public health burden associated with alcohol use is substantial. The World Health Organization (WHO) [3] estimates that alcohol contributes to 2.6 million deaths annually worldwide. Alcohol consumption is associated with more than 200 diseases and injuries, including liver cirrhosis, cardiovascular diseases, cancers, road traffic injuries, interpersonal violence, and mental health disorders [5-7]. The burden is especially pronounced in low- and middle-income countries, with the WHO African Region recording some of the highest alcohol-attributable mortality and disability-adjusted life year rates globally [8]. Research further shows strong bidirectional relationships between alcohol use and mental health conditions such as depression and anxiety, where psychological distress may contribute to alcohol use while heavy drinking simultaneously worsens emotional well-being [9].

In Ghana, these global patterns are reflected in national trends. WHO [3] estimates total alcohol per capita consumption at 4.5 liters of pure alcohol annually. Alcohol use remains a significant public health concern, particularly among adults aged 15 years and older, with consumption patterns generally higher among men than women. According to a GhanaWeb [10] report citing the Mental Health Authority of Ghana, 3765 alcohol-related mental health cases were recorded in 2023. Kyei-Gyamfi et al [11] note that among adolescents, alcohol is the most commonly used substance. They link early alcohol use to peer pressure, curiosity, and coping motives. These trends occur alongside rapid urbanization and changing leisure cultures [12,13].

Alongside this epidemiological evidence of harm, alcohol is often represented as an ordinary and even beneficial part of life. Research from high-income settings shows that advertising, entertainment media, and digital communication frequently portray drinking as integral to sociability, emotional regulation, relaxation, and pleasure [14,15]. With the rise of social media, these representations have become increasingly participatory and peer-driven. Studies of user-generated content on platforms such as Facebook, X (formerly known as Twitter), Instagram, and TikTok suggest that alcohol is commonly depicted as humorous, socially expected, and emotionally rewarding [16,17]. Social media platforms, therefore, do not simply reflect attitudes toward alcohol. They also shape how drinking is normalized, justified, and embedded within social interactions.

Despite the global reach of social media platforms, most research on alcohol-related digital discourse remains concentrated in Western societies [18]. African contexts remain comparatively underexplored, even though social media is now deeply embedded in everyday communication and social interaction across the continent. This gap is significant because the meanings attached to alcohol and the discursive resources used to justify its consumption are shaped by local sociocultural contexts. However, there is limited research examining how Ghanaian social media users themselves construct alcohol in relation to emotional coping and social interaction. Understanding these constructions is important for digital health communication because social media increasingly shapes how health-related behaviors, coping practices, and everyday forms of self-care are interpreted. Without attention to these lived and digitally circulating meanings, public health communication risks becoming detached from the social realities through which alcohol use is legitimized and sustained. This study addresses this gap by asking: How is alcohol represented in Ghanaian social media discourse as a resource for emotional coping and social interaction?

### Theoretical Grounding: Social Constructionism and Discourse Theory

This study is grounded in social constructionism, which provides its overarching theoretical orientation. Social constructionism posits that social realities, health behaviors, and risk perceptions are not objective, preexisting facts waiting to be discovered, but are produced, negotiated, and maintained through social interaction [19,20]. From this perspective, public understandings of alcohol use, including concepts of pleasure, intoxication, coping, and harm, are not simply fixed biological or psychological realities. Instead, they emerge through culturally and historically specific ways of talking about drinking, risk, friendship, and leisure [19]. The significance of alcohol, therefore, extends beyond its physiological effects to include the meanings attached to drinking through everyday communication. This perspective shifts the focus from measuring individual psychological motivations to examining how particular understandings of alcohol are constructed through language and interaction.

Discourse theory provides the broader analytical approach through which these constructions are examined. Within discourse-oriented research, language is understood as constitutive rather than descriptive; it does not simply reflect an external social reality but actively helps create, sustain, and contest meanings [4,21,22]. Discourses can be understood as broader ways of organizing language and meaning around particular subjects, shaping how those subjects can be understood and discussed within specific contexts [23]. Through discourse, behaviors may be normalized, justified, challenged, or stigmatized. Within public health, competing discourses therefore shape how health, responsibility, risk, and acceptable behavior are understood [24]. While clinical and epidemiological discourses often frame alcohol through pathology, harm, and risk, popular and commercial discourses may construct drinking through narratives of vitality, emotional relief, social connection, and everyday usefulness. These competing ways of talking about alcohol help explain how drinking can remain socially meaningful even where its health risks are well documented.

In the Ghanaian context, these discursive processes intersect with local economic, cultural, and regulatory conditions. Alcohol has long been intertwined with social and ritual practices, ranging from traditional libation ceremonies to the widespread availability of commercial beverages, locally distilled spirit known as akpeteshie, and the marketing of products such as bitters and sachet spirits [12,25,26]. These cultural and commercial contexts are important to this study because they shape the meanings available for talking about alcohol. Examining alcohol-related discourse in Ghana, therefore, involves more than identifying individual attitudes toward drinking. It involves examining how alcohol is made meaningful and how some forms of consumption are presented as legitimate, desirable, or necessary within particular social contexts.

Interpretative repertoires provide the specific analytical tool used in this study to examine these recurring constructions. They are recurrent, flexible, and culturally familiar systems of terms, descriptions, and figures of speech used to characterize, evaluate, and explain social phenomena [4]. They provide users with shared linguistic resources for constructing versions of reality, explaining actions, and managing how their drinking is understood in interaction [27]. Previous studies demonstrate how people mobilize these repertoires to construct health behaviors, illness experiences, and moral identities [27]. Within discussions of alcohol use, interpretative repertoires can depict drinking as essential for relaxation, handling emotional stress, a sign of sociability, or part of daily coping habits. In this study, identifying these repertoires enables the analysis of recurring ways in which alcohol is constructed as useful, legitimate, and socially meaningful.

Social media provides an appropriate setting for examining these constructions because meanings are produced and negotiated through everyday interaction. Facebook and TikTok bring together peer commentary, commercial messages, humor, storytelling, and locally grounded forms of expression. In Ghana, users combine global social media trends with expressions in local languages and Ghanaian Pidgin English (GhPE), an English-lexified contact variety widely used in informal communication [28]. The analysis of interpretative repertoires in these spaces allows the study to examine recurring constructions of alcohol in naturally occurring digital communication. The focus is not only on user-generated content about alcohol but also on the discourse that makes drinking seem appropriate, beneficial, desirable, or socially expected.

## Methods

### Research Design

This study adopts a qualitative, exploratory design grounded in social constructionism and follows the discourse-analytic tradition associated with Potter and Wetherell [4] and Wetherell [22]. Within this tradition, discourse is understood as language-in-use that is action-oriented and constitutive: it participates in constructing, negotiating, and making meaningful social realities, identities, evaluations, and forms of action. The analysis is therefore interpretive rather than representative. Consistent with contemporary discourse studies of digital communication, discourse in this study is not restricted by text length or the presence of extended conversational exchange. Social media posts and comments are treated as discursive acts because they are produced for public audiences and perform social actions such as justifying behavior, expressing emotions, constructing identities, negotiating relationships, and circulating culturally shared understandings. Although many social media contributions are brief, they remain analytically valuable because they draw upon broader cultural resources and shared systems of meaning [29]. The study is therefore concerned not with whether alcohol actually produces the effects attributed to it by users, but with how alcohol is discursively constructed as meaningful, useful, desirable, therapeutic, or socially significant within everyday online interactions. It seeks to identify the shared interpretative repertoires through which alcohol is constructed as a resource for emotional coping and social interaction in Ghanaian social media.

### Data Source and Platform Selection

Data consist of publicly available user-generated content, including posts and comments, from 2 widely used platforms in Ghana: Facebook and TikTok. Facebook was selected for its extensive user base across multiple demographic groups in Ghana, its support for longer-form messages and community group discussions, and its rich, multimodal content, including text, images, and video. Although the broader social media environment contained visual content such as photographs, videos, emojis, Graphics Interchange Format files, and stickers, this study focused exclusively on textual discourse. During data preparation, all nontextual elements were excluded from the analytical dataset. That is, the analysis was limited to posts and comments and did not examine visual representations of alcohol. This decision ensured analytic consistency and allowed the study to focus specifically on how alcohol was constructed through language. TikTok, which has rapidly grown in popularity in West Africa, was included to capture shorter, performative, and humor-driven content circulating through sounds, hashtags, and challenges. These 2 platforms offered complementary windows into different interactional styles. Facebook enabled analysis of more elaborate narrative accounts and exchanges, while TikTok provided access to condensed, affectively charged, and memetic expressions. These affordances shape how alcohol is represented and the intensity of affective expression in messages.

Only textual content that was publicly visible without login or membership was included. No attempts were made to access locked accounts, individual demographic parameters (such as age, gender, or social status of commenters) were not collected, and no interactions with users occurred. While using publicly available data does not require formal institutional ethics approval, ethical considerations remained central to the research process. In line with established guidelines for internet-mediated research [30,31], all usernames were deleted, and care was taken to minimize potential harm.

### Sampling and Search Strategy

A purposive, keyword-driven sampling strategy was used to identify messages in which alcohol was explicitly linked to emotional coping, mood regulation, or social interaction. Prior to data collection, 2 bachelor-prepared research assistants conducted a month of preliminary ethnographic observation of Ghanaian social media spaces to become familiar with common phrasing patterns. This immersion informed the development of a search lexicon that included English terms (eg, alcohol, drink, drunk, beer, and wine), Ghana-specific beverages and brand names (eg, akpeteshie, pito, palm wine, Kasapreko, Adonko, and Alomo Bitters), and colloquial expressions (eg, “I need a drink,” “make I drink,” “broken heart medicine,” “body dey pain me,” and “man must chill”). Searches were conducted using the native search functions of Facebook and TikTok, supplemented by manual scrolling through relevant pages. To ensure comprehensive coverage while prioritizing recency, data collection began with the most recent year (2025) and moved backward to 2018. This backward sampling approach enabled the identification of current discursive patterns while also assessing their continuity over time.

The initial search yielded 266 messages that made some reference to alcohol. These were then screened against inclusion and exclusion criteria. For inclusion, messages were required to be in English, GhPE, or code-switched constructions involving a Ghanaian language and to make a direct discursive connection between alcohol and an emotion, mood state, or interpersonal context. Additionally, the messages had to be clearly produced by a user based in Ghana or by a user participating in a recognizably Ghanaian conversational community, a judgment made through indicators such as geotags, self-reported location, linguistic markers, and references to Ghana-specific places, events, and public figures. Messages were excluded if they were purely commercial promotions that lacked user commentary or interaction, duplicate content, or news headlines shared without personal commentary.

After this screening process, 189 messages were retained for analysis. The corpus comprised 153 Facebook messages and 36 TikTok messages, of which 41 were original posts and 148 were user comments. The predominance of comments reflects the study’s interest in naturally occurring public discussions surrounding alcohol-related content. These comments typically appeared in response to birthday celebrations, party announcements, alcohol advertisements, public health messages, and other alcohol-related posts. Therefore, much of the dataset was situated within interactional contexts. As will be shown in the next section, examples included discussions surrounding palm wine consumption, the use of bitters such as Alomo Bitters, and references to alcohol as a remedy for physical or emotional distress. These discussions provided additional insight into how alcohol-related meanings are shared and reinforced on social media.

In instances where a message was lengthy or addressed multiple topics, only the portion that directly concerned alcohol in relation to emotion or social interaction was extracted, and the extraneous content was not coded. While the analysis dataset is modest in size, it is consistent with qualitative discourse-analytic research, where depth of interpretation is prioritized over breadth. Sampling continued until no substantively new discursive patterns were observed, suggesting that key repertoires had been sufficiently captured [32].

### Analytical Procedure

The analysis followed an iterative, interpretive process informed by the discourse-analytic procedures outlined by Potter and Wetherell [4] and Wiggins [27]. The authors (RGAT, TT, and MKG) began by repeatedly reading the dataset to become familiar with its content. During this initial familiarization stage, each message was assigned a unique alphanumeric identifier consisting of the prefix “P” (denoting a public message) followed by a sequential number (eg, P1 and P2) to facilitate data management, coding, and retrieval. The authors paid attention to recurring phrases, metaphors, and the social actions performed in messages (eg, complaining, bonding, joking, and self-medicating) and recorded their initial observations. These identifiers were subsequently used throughout the analysis and the Results section to reference specific data excerpts while maintaining the anonymity of individual contributors.

The analysis moved from preliminary observations to the identification of interpretative repertoires. An interpretative repertoire is understood as a broadly shared, relatively coherent way of talking about a phenomenon that furnishes speakers with a range of linguistic resources for constructing it [22]. Through iterative grouping and refinement of codes, the team identified a set of repertoires that constructed alcohol, for example, as a therapeutic agent, as a social connector, and as a routine mood regulator. These repertoires were then compared across the dataset to identify similarities and differences in how alcohol was constructed. Table 1 illustrates this analytical progression using selected examples from the corpus, showing how recurring textual features were developed into descriptive codes and subsequently interpreted as elements of broader interpretative repertoires. The examples are illustrative rather than exhaustive and are intended to make transparent how the analytical categories were developed from the data.

**Table 1:** Illustrative progression from data extracts to interpretative repertoires.

Representative extract	Initial code	Interpretative repertoire	Analytical category
“You are not in Alcoholism. Your own na to cool your mind sharp”	Alcohol relieves stress	Alcohol as coping resource	Alcohol as emotional repair
“I’m drinking alcohol now because cocoa prices have fallen”	Drinking as response to economic distress	Alcohol as coping resource	Alcohol as emotional repair
“He was with me when I had a broken heart”	Alcohol as emotional companion	Alcohol as coping resource	Alcohol as emotional repair
“We club with club beer to socialise and brainstorm”	Alcohol facilitates interaction	Alcohol as social bonding	Alcohol as social connector
“No matter the fyt, we still sit and drink Guinness and Smirnoff together”	Drinking sustains friendship	Alcohol as social bonding	Alcohol as social connector
“Palm wine is the only authentic drink our gods approve”	Alcohol linked to tradition and spirituality	Alcohol as cultural belonging	Alcohol as social connector
“Alomo dey for every mood”	Alcohol improves mood	Alcohol as emotional regulation	Alcohol as everyday mood regulation
“One sip and you’re already feeling like you’re at a village festival, good vibes, sweet taste, zero stress”	Alcohol produces positive affect	Alcohol as emotional regulation	Alcohol as everyday mood regulation
“Obuase Bitters ... strengthens your immunity and powers your stamina”	Alcohol framed as health-enhancing	Commercial-health discourse	Commercial-lay hybrid discourse
“Palm wine contains yeast ... it relieves stress and is good for pregnant women”	Personal testimony used to support health claims	Commercial-health discourse	Commercial-lay hybrid discourse

Alongside the mapping of repertoires, the analysis examined how posts positioned speakers and audiences. For each repertoire, we examined the identities being claimed or ascribed, such as the person seeking emotional relief, the loyal friend for whom drinking together is a sacred ritual, and the hardworking individual who has earned the right to relax. We also considered how relationships with audiences were constructed through shared cultural knowledge, humor, and social expectations. Given that the data were drawn from 2 platforms with different affordances, the analysis remained sensitive to platform context without treating them as separate datasets. Facebook content often permitted more extended narrative development and was frequently accompanied by photographs of drinks or drinking scenes that reinforced their textual meanings. TikTok content, in contrast, tended toward brevity, irony, and memetic humor, with text overlaid on short video clips and the creative use of platform-specific features such as soundtracks and duets. While the study did not analyze visual content, it remained attentive to how each platform’s communicative affordances shaped the style, tone, and structure of textual contributions. These variations were treated as complementary expressions within a shared digital environment in which alcohol-related meanings were produced and circulated.

The identified repertoires were then examined in relation to the broader functions they served within the dataset. These included alcohol as a social connector, covering friendship, leisure rituals, and communal identity; alcohol as emotional repair, encompassing references to stress, heartbreak, and bodily discomfort; and alcohol as everyday mood regulation, linked to routine leisure and the transition into weekend time. Commercial and promotional messages that had been shared, commented on, or repurposed by ordinary users were also analyzed for how they interfaced with and reinforced these lay repertoires. Throughout the process, the authors engaged in peer debriefing, maintained a reflexive audit trail, and actively tested interpretations against deviant cases in order to enhance the rigor and credibility of the analysis.

All authors were familiar with Ghanaian social media culture and understood local drinking practices. This helped them interpret the data more deeply and accurately. However, because of this familiarity, there was also a risk of overlooking assumptions or treating certain meanings as obvious. To address this, the authors continually reflected on how their backgrounds and perspectives might influence their interpretations throughout the analysis. The authors also remained attentive to the performative nature of social media discourse, where humor, exaggeration, and promotional language may shape how alcohol is represented. Particular care was taken not to infer audience beliefs or broader social consensus from individual messages. Instead, interpretations focused on how alcohol was presented, justified, normalized, or made meaningful within the messages themselves, rather than treating individual contributions as evidence of what social media users generally believed. Thus, the analysis focused on the social and rhetorical functions performed through constructions of alcohol use.

### Ethical Considerations

All data were collected from publicly accessible social media spaces. Thus, ethical clearance was not obtained from any institution. However, in line with the Association of Internet Researchers’ guidelines [30], all usernames, profile images, and other identifying markers have been removed from the excerpts reported in this paper to protect anonymity and minimize any risk of harm. No direct interaction with users occurred at any stage of the research.

## Results

### Overview

Of the 189 messages analyzed, 3 interpretative repertoires were identified through which alcohol was discursively constructed in Ghanaian social media discourse. These repertoires portrayed alcohol as a resource for emotional repair, a social connector, and a means of routine mood and bodily regulation. The analysis focused on how particular ways of talking about alcohol make drinking appear meaningful, legitimate, desirable, or socially appropriate, without assuming that individual messages directly reflect personal beliefs or behaviors. A fourth cross-cutting repertoire, termed commercial-lay hybrid discourse, shows how promotional language and everyday user narratives intersect to reinforce these constructions. In the excerpts that follow, the textual contents are reproduced verbatim except where usernames and other potentially identifying details have been removed or altered to protect privacy.

### Alcohol as Emotional Repair

The first major repertoire positioned alcohol as an accessible, effective remedy for a range of emotional discomforts. Within this repertoire, several recurring discursive patterns were identified: everyday stress, heartbreak, and romantic loss.

#### Everyday Stress and Mental Pressure

In the following, alcohol is framed as a necessary tool for calming the mind.

Chairman. You are not in Alcoholism. Your own na to cool your mind sharp.

P1

P1 pushes back against the stigma of being labeled an “alcoholic” by drawing a line between problematic drinking and a purposeful act. It performs identity work by resisting the potentially stigmatizing label of “alcoholic.” The commenter constructs drinking as a purposeful and rational response to emotional strain, positioning alcohol use as a form of active emotional management rather than as evidence of dependency or loss of control. The phrase “cool your mind” (figurative expression in GhPE) draws on a culturally familiar repertoire in which emotional distress is framed as something that can be actively managed through drinking.

Similarly, economic distress was explicitly named as a trigger for drinking: “I’m drinking alcohol now because cocoa prices have fallen” (P56).

The blunt causal structure presents alcohol use as an immediate and natural response to economic distress. The comment links an economic issue (falling cocoa prices) to an individual coping practice (drinking). By making this connection directly, the commenter constructs alcohol use as a commonsense response to economic disappointment, without presenting the drinking itself as requiring explanation or justification. The brevity of the statement reinforces this construction by treating the connection between economic hardship and alcohol consumption as self-evident within the communicative context. Alcohol is thus positioned as a way of responding to the pressures of economic hardship.

#### Heartbreak and Romantic Pain

The metaphor of alcohol as “medicine” for a broken heart was a highly visible repertoire. This trope drew on both global digital culture and local bitters brands that already carry medicinal connotations.

Please help me say thank you to this wonderful friend of mine. He was with me when I had a broken heart, when I lost my mum he was there and also when Business was like a Methodist hymn he was with me. To the Ultimate in beer GULDER aka Booooosu.

P15

In P15, a beer brand is anthropomorphized as a steadfast companion through bereavement, romantic pain, and business stagnation. Through personification, the beer brand is portrayed as a dependable companion that offers emotional support across various life difficulties. It is clear that the poster uses a range of symbols and portrays alcohol as a symbol of loyalty, companionship, and emotional connection.

... it’s a good appetizer for people recovering from sickness and people who have lost appetite for food due to broken heart and love related complications ...

P114

Here, alcohol is described as helpful both for “people recovering from sickness” and for those who have “lost appetite for food due to broken heart and love-related complications.” By placing emotional pain alongside physical illness, P114 treats heartbreak as a condition with bodily consequences, particularly loss of appetite.

### Alcohol as a Social Connector

Another repertoire constructed alcohol as central to the formation and maintenance of social relationships. Drinking was associated with friendship, celebration, communal identity, and cultural belonging.

#### Drinking as the Substance of Friendship

No matter the fyt, we still sit and drink Guinness and Smirnoff together ... d love is deep ruff ... Guinness nd Smirnoff is bae ... try it.

P45

P45 constructs shared drinking as a relational practice through which friendship is maintained despite interpersonal conflict. The poster foregrounds the continued ability to drink together as evidence of an enduring social bond. The exaggerated affectionate expression “d love is deep ruff” (the love is very strong) and the description of Guinness and Smirnoff as “bae” (an affectionate term meaning beloved) further reinforce this construction by presenting the drinking relationship in terms of closeness, affection, and familiarity. Alcohol consumption, therefore, functions as a symbolic marker of solidarity and relational continuity.

The 3 things in life that are dearest to me: My wife, Star beer, and fufu!

P38

By placing beer alongside a spouse and fufu, a culturally valued staple food, the poster elevates alcohol to an important, emotionally meaningful component of everyday life. He normalizes alcohol by embedding it within familiar narratives of comfort, pleasure, and fulfillment.

#### Drinking Rituals, Communal Identity, and Call-and-Response

In my community ... Sam1 will shout ULTIMATE nd de other will respond PARADISE ... This is written on this faithful companion We also got love 4 it too.

P16

Call-and-response exchanges are a familiar communicative practice in many Ghanaian social settings. The use of the call-and-response pattern “ULTIMATE” ... “PARADISE” around the alcohol brand transforms alcohol consumption into a participatory communal performance rather than an individual activity. Participation signals familiarity and belonging within the community.

Apobona Fatawu we club with club beer to socialise and brainstorm 1,2 and a mini for the road.

P2

Here, drinking is directly linked to social interaction and conversation. The reference to “socialize and brainstorm” constructs alcohol as a facilitator of interpersonal engagement and collective reflection. In Ghanaian colloquial use, “1,2” conveys the idea of activities unfolding continuously or one after another, while “a mini for the road” refers to a final small drink before departure. P2 thus normalizes drinking as an integral part of extended social interaction.

#### Celebrations and Life Course Events

Happy birthday to you my day one brother. May you live to see your great grandchildren ... 365 days nor bi easy my brother so enjoy till you booze.

P37

The directive “365 days nor bi easy my brother so enjoy till you booze” (365 days is not easy, my brother, so enjoy till you are drunk) portrays intoxication as an appropriate reward for enduring the difficulties of everyday life. Here, drinking is presented as part of marking survival, celebration, and personal milestones.

Your Valentine isn’t complete without Kasapreko in the mix because, every great #love story deserves a bold taste, that lingers.

P46

This promotional post links alcohol to romantic fulfillment by suggesting that celebrations of love are incomplete without drinking Kasapreko. Alcohol is positioned as something that enhances intimacy and emotional experience.

#### Palm Wine, Tradition, and Cultural Authenticity

Palm wine is the only authentic drink our gods approve. It has to be taken fresh from the lips of the palm tree early in the morning. We travel from the cities to the villages just for Palm wine. The best we can do is freeze some quantities for family and friends in the city ...

P28

This comment portrays palm wine as more than an alcoholic beverage. It is presented as culturally authentic and closely associated with tradition and rural life. The phrase “our gods approve” invokes a cultural repertoire that links the drink to long-standing spiritual and communal practices, such as libations and rituals. The effort involved in transporting and freezing palm wine for relatives further frames it as a socially meaningful commodity through which care and social connection are expressed.

In Ghana, palm wine or nsafufuo is a traditional drink made from fermented palm sap. It’s central to social and spiritual life, known as the drink of the gods.

P19

This comment constructs palm wine as more than an alcoholic beverage. By describing it as “the drink of the gods,” the poster draws on a culturally recognizable discourse that links palm wine to spirituality and tradition, as shown in P28. The comment thus positions palm wine as a culturally valued substance whose significance extends beyond consumption.

### Alcohol as a Resource for Everyday Mood and Bodily Regulation

Beyond emotional distress or social occasions, a third repertoire positioned alcohol as part of the routine management of everyday mood, especially in relation to leisure, relaxation, weekend enjoyment, and bodily well‑being.

#### Leisure, Relaxation, and Emotional Enhancement

Alomo dey for every #mood ... Don’t be left out this weekend.

P3

This post projects the alcohol brand as a flexible emotional resource capable of fitting any affective circumstance. By claiming that “Alomo dey for every #mood” (Alomo is available for every mood), the poster presents drinking as a broadly applicable strategy for managing everyday emotional states. The reference to the weekend also connects drinking to participation in shared leisure culture.

What better way to slide into the TGIF mood than with a bottle of Alomo Bitters and a fun emoji game?

P10

Here, alcohol is shown as a resource that aids the shift from a busy workweek into relaxation and enjoyment. The phrase “slide into the TGIF mood” depicts relaxation as a positive emotional change, with alcohol serving as a facilitator of this transition. Consequently, drinking is portrayed as a natural transition from work duties to leisure.

I kept it simple ... grilled a full chicken, paired it with an Alomo Bitters cocktail lol. Lazy day but still premium.

P77

In P77, alcohol is constructed as a resource for enhancing ordinary leisure experiences. The contrast between a “lazy day” and a day that is “still premium” positions the Alomo Bitters cocktail as the element that elevates an otherwise unremarkable occasion. Drinking is therefore presented not as a response to distress but as a means of adding value, enjoyment, and distinction to everyday life.

Obuasi bitter can make ur day.

P13

One sip and you’re already feeling like you’re at a village festival good vibes, sweet taste, zero stress.

P49

Through expressions such as “make ur day” and “one sip and you’re already feeling,” both P13 and P49 attribute some transformative powers to Obuase Bitters. They present positive emotional states as immediate and attainable through consumption. That is, they normalize drinking as an ordinary means of regulating affect and generating pleasurable experiences.

Some users also drew on the language of hormones and emotional balance to justify drinking as a form of self-regulation.

So no benefits from drinking responsibly? After all me here needs that happy hormones to release stress, wat do I gain from stress n sad hormones.

P52

The commenter rhetorically questions a public health message about “drinking responsibly” by emphasizing alcohol’s perceived emotional benefits. The phrase “happy hormones” draws on quasi-biomedical language to construct drinking as a legitimate and even rational response to stress. By contrasting “happy hormones” with “stress” and “sad hormones,” the commenter mobilizes a health-oriented vocabulary that frames alcohol consumption as beneficial to emotional well-being rather than as a potential health risk.

I will rather stay away from women than alcohol. Alcohol releases hormones and women impact pains.

P63

In this context, alcohol is compared to intimate relationships. While relationships are associated with “pains,” alcohol is depicted as a more dependable source of emotional support and steadiness. Just as shown in P52, here, the commenter moves beyond pleasure-seeking reasons and reframes alcohol consumption as a reasonable strategy for maintaining emotional well-being.

#### Bodily Discomfort and Functional Use

Some messages presented alcohol, particularly herbal bitters and palm wine, as a practical remedy for bodily discomfort, especially appetite loss and digestive problems.

After taking Adonko bitters I’m able to finish the Ghana fufu with light soup.

P42

This is appetite stimulant.

P5

It helps my bowel movement.

P22

Yebu didi (Akan) “We get drunk to eat.”

P157

These comments link drinking to eating, digestion, and bodily comfort. They present alcohol not mainly as recreation but as a tool for daily well-being. Drinking is portrayed as a practical way to cope with appetite loss and discomfort. It is seen as a normal part of daily health habits, which helps to normalize its use and makes it more acceptable.

A similar functional logic also appeared in relation to cognitive performance and studying.

But please what about we those will take before we can learn if i have exams i have to take before everything.

P98

In P98, alcohol is described as necessary for studying, especially during examinations. This challenges conventional assumptions that alcohol impairs cognitive performance. Through this reversal, alcohol is discursively reconstructed as a facilitator of concentration and mental readiness rather than a potential obstacle to learning.

### Commercial-Lay Hybrid Discourse

The final repertoire captures the overlap between promotional messaging and everyday user narratives across the 3 repertoires presented earlier. In the examples that follow, it is clear that commercial actors and ordinary users are not engaged in separate meaning-making processes. Both contribute to a shared discursive environment in which alcohol is linked to vitality, immunity, and emotional fulfillment. As these meanings circulate across posts, comments, testimonials, and promotional messages, the distinction between advertising claims and everyday knowledge becomes increasingly blurred.

Obuase Bitters: crafted from pure herbs, the enduring alcoholic beverage that strengthens your immunity and powers your stamina. Try it out and thank Dr Amo Collins later.

P14

Here, the poster endorsed Obuase Bitters by using advertising language with the authority attached to the company founder, “Dr Amo Collins.” The references to “pure herbs,” “immunity,” and “stamina” frame the product in therapeutic terms. It blurs the boundary between commercial promotion and health-oriented advice.

Paim[SIC] wine containes[SIC] yeast it even cure’s fewer[SIC] for one to believe what am saying watch what you are eating or drinking, it relief you from stress and is good for pregnant women cause it increase breast milk, am witness to it.

P61

This relies on personal testimony to construct palm wine as medically beneficial. The commenter attributes multiple functions to palm wine, including fever relief, stress reduction, and increased breast milk production. The phrase “am witness to it” positions lived experience as evidence.

Get Smirnoff Ghana Smirnoff pineapple punch with a touch of chicken wings this hot afternoon and trust me ya will be in heaven.

P94

P94 frames alcohol as a source of pleasure and sensory enjoyment. The combination of the drink with food and the relaxed setting of a “hot afternoon” constructs drinking as part of an enjoyable leisure experience.

Opening Nkulenu Palm Wine may cause sudden happiness, loud laughter, and unnecessary enjoyment.

P48

This post playfully imitates the language of product warnings by using the phrase “may cause.” However, instead of warning about harm, the post lists desirable emotional outcomes such as “happiness,” “loud laughter,” and “enjoyment.” The reversal presents the pleasurable effects of alcohol as desirable and socially valued outcomes of drinking.

## Discussion

### Principal Findings

This study examined how alcohol is represented as a resource for emotional coping and social interaction in Ghanaian social media discourse. The findings reveal a rich discursive landscape in which alcohol is constructed as an accessible and legitimate resource for managing emotional, social, and bodily concerns. Within the identified repertoires, alcohol is seen not only as a beverage but also as a culturally significant means of managing stress, heartbreak, physical discomfort, social duties, and leisure activities. The analysis shows how social media users draw on shared interpretive tools to normalize drinking, justify its use, and weave it into everyday ideas of well-being, friendship, and cultural identity. A commercial-lay hybrid discourse illustrates how promotional claims and everyday user narratives converge to reinforce these constructions.

The findings extend existing scholarship on alcohol and emotion regulation, which has predominantly approached the issue through psychological or epidemiological lenses, by showing how coping itself is actively constructed in everyday digital discourse. In these messages, drinking is not simply reported as behavior. It is rhetorically organized to accomplish social actions such as justifying relaxation (You are not in Alcoholism), fostering resilience through humor (broken heart medicine), building solidarity through shared cultural language (ULTIMATE ... PARADISE), and claiming a culturally sanctioned form of self-care. More specifically, the findings demonstrate how discourse functions as a mechanism through which particular coping practices become intelligible and socially acceptable. Through humor, testimony, personification, and appeals to common sense, users construct alcohol consumption as an appropriate response to emotional difficulty. From this perspective, alcohol can be understood as a culturally available resource through which individuals make sense of and manage emotional experience. This aligns with social constructionist approaches that treat language as constitutive [4,33].

These coping constructions are intertwined with the ways alcohol is used to organize social life and relational identity. The findings confirm and culturally specify the well-documented role of alcohol in social bonding [2,18]. They also support existing work, suggesting that alcohol is frequently represented as a medium through which intimacy, celebration, and relational closeness are performed and maintained [34]. Messages that framed Valentine’s celebrations or birthdays as incomplete without alcohol illustrate how drinking becomes symbolically tied to affection, solidarity, and shared emotional experience. However, the data highlight nuances that are less visible in Western contexts. The personification of beer brands as companions in moments of grief or business difficulty (P15) extends beyond conventional constructions of drinking as a shared social practice. Similarly, call-and-response drinking chants (P16) and the practice of traveling to rural areas to obtain fresh palm wine for sharing with family (P28) situate alcohol within broader traditions of orality, communal care, and rural-urban kinship. In this sense, drinking is not merely a leisure activity but an enactment of relational obligation and cultural belonging. From a discourse-theoretical perspective, these constructions do more than describe existing social relationships. They help reproduce and sustain particular understandings of friendship, loyalty, reciprocity, and communal participation. Alcohol becomes a symbolic resource through which social identities are performed and affirmed [4,22,34].

Alcohol is also consistently integrated into ordinary life as a routine means of mood regulation and transition into leisure. Messages that frame drinking as appropriate for “lazy days,” weekends, or any emotional state illustrate how alcohol becomes normalized as a resource for managing everyday affect. In the Ghanaian context, the weekend discourse is particularly salient as it draws on both the cultural familiarity of communal end-of-week gatherings and the globalized TGIF (Thank God It’s Friday) ethos. Within this framing, alcohol is often constructed as the means to “slide into” weekend mood, positioning it as a benign and, at times, necessary boundary marker between labor and restoration. This reflects broader processes of normalization, in which repeated associations render alcohol use routine, expected, and socially unremarkable, particularly within digitally mediated leisure cultures [17,35]. The significance of this repertoire lies in how these ordinary references make drinking part of everyday emotional life without requiring explicit advocacy for alcohol use.

The findings related to appetite stimulation, digestion, and studying further illustrate how alcohol is constructed as a functional resource embedded in everyday life. The repeated emphasis on appetite suggests that these messages construct eating ability as closely linked to bodily comfort and well-being. Similarly, messages describing alcohol as necessary for studying position drinking as cognitively enabling and supportive of concentration. These constructions challenge dominant public health narratives that primarily associate alcohol with impairment, irresponsibility, and loss of control [36]. They align more closely with culturally grounded understandings, in which alcohol is constructed as a functional resource capable of addressing practical bodily and emotional concerns [37]. The repertoire, therefore, expands the perceived usefulness of alcohol beyond recreation and positions drinking as compatible with productivity, health, and everyday competence.

A broader commercial-lay hybrid discourse emerges, in which promotional language, health claims, and everyday user talk become increasingly difficult to distinguish. Messages that frame bitters as immune boosters or palm wine as medically beneficial are not just treated as external marketing messages. They are taken up, circulated, and reformulated within peer communication. Through these processes, alcohol becomes associated with well-being, vitality, and emotional restoration. This convergence illustrates how commercial alcohol promotion draws legitimacy from locally meaningful health beliefs, while everyday users actively reproduce and adapt these claims in ways that blur boundaries between consumption, care, and cultural knowledge. It is noteworthy that alcohol promotion on social media operates through participatory, peer-driven forms of communication rather than solely through formal advertising. Users do not simply consume marketing messages; they actively reinterpret, personalize, and redistribute them through humor, testimony, lifestyle performances, and everyday interaction. In this sense, social media users function simultaneously as consumers and informal promoters of alcohol-related meanings, reflecting broader dynamics of prosumer culture and participatory advertising within digital environments. The persuasive force of these messages may partly derive from their appearance as authentic peer-to-peer communication instead of institutional advertising. This finding highlights the need to reconsider how alcohol promotion is regulated in Ghanaian digital spaces, where existing advertising codes may not fully address the participatory, rapidly evolving nature of social media promotion [13,38]. Traditional regulatory approaches are typically designed to monitor formal advertising. However, the findings suggest that alcohol-related promotional messages increasingly circulate through user-generated content, testimonials, humor, and peer-to-peer sharing. This creates regulatory challenges because many such messages fall outside conventional definitions of advertising. Future policy discussions should therefore consider how public health protections can be extended to participatory digital spaces without undermining legitimate forms of online expression.

In all, the findings show that alcohol in Ghanaian social media discourse was more often constructed as a multifunctional resource embedded in emotional, social, and cultural life than as a risky substance. Across the repertoires, therapeutic, relational, and commercial meanings intersect to make drinking intelligible, useful, and socially acceptable in everyday life. These constructions have implications for health communication because they show that alcohol is normalized not only through promotion, but also through ordinary practices of coping, social connection, and leisure.

### Implications

Comments referring to alcohol as “medicine” and as a resource for socialization and even “brainstorming” pose a significant challenge for health promotion. Such messages frame drinking as beneficial to health and socially normative, thereby establishing a discursive environment, in which messages emphasizing harm or promoting abstinence may encounter difficulties in gaining traction. Effective health communication in this context may therefore need to engage with, rather than dismiss, these everyday meanings. This includes developing alternative, culturally resonant scripts for coping and connection that acknowledge the emotional and social functions alcohol currently fulfills, while gradually reframing these functions in healthier directions.

Understanding diverse social media constructions of alcohol may also help public health practitioners and clinicians develop more targeted approaches to prevention and intervention [39,40]. The distinction observed in the present data between alcohol framed as a means of managing negative experiences, such as stress or heartbreak, and alcohol framed as a means of enhancing social experiences has parallels with established research on drinking motives, including relief and reward motives [39,40]. These motivational patterns are clinically relevant, as research suggests that relief-oriented drinking may be associated with greater difficulties during treatment than drinking motivated primarily by social or enhancement motives [39,40]. However, the present study does not establish that users who describe alcohol in these terms meet criteria for alcohol use disorder or correspond to particular clinical subtypes.

This distinction is important for interpreting the implications of the findings. Social media messages that construct alcohol as “medicine” may provide useful entry points for health communication, especially around coping with stress and emotional challenges. Conversely, messages that link drinking to friendship and social enjoyment may require distinct strategies. Instead of relying on generic warnings about alcohol-related harm, campaigns could co-design messages with communities that recognize the social and emotional roles attributed to drinking while also suggesting practical alternatives that can be integrated into the same digital environments. Brief motivational interventions that address specific reasons for drinking have been shown to be effective, particularly among individuals whose alcohol consumption is driven by social or enhancement motives [41]. These strategies highlight the importance of moving away from a one-size-fits-all approach toward health communication that considers the diverse meanings of alcohol use in everyday life.

### Limitations

Several limitations should be noted. The dataset, while rich, is drawn from public messages and does not capture private messaging or offline talk, where different discursive conventions may apply. Since the messages were publicly accessible, users might also have filtered or curated what they posted in ways that differ from communication intended for smaller or more private audiences. The analysis was also restricted to textual posts and comments. Images, videos, emojis, Graphics Interchange Formats, and other visual elements were excluded from the analytical corpus. Consequently, the study does not capture the multimodal ways in which alcohol may be represented across social media platforms. In addition, the use of reverse chronological sampling, beginning with the most recent messages and moving backward, prioritized recency over equal temporal coverage. Although this approach enabled the identification of contemporary discursive patterns and an assessment of their continuity over time, it may have overlooked earlier or less persistent forms of discourse that were not visible in more recent periods. The dataset contained both original posts and user comments, but the study did not systematically compare discursive patterns across these 2 forms of participation. Future research could examine whether alcohol-related meanings are constructed differently by content creators and audience members.

The analysis did not differentiate between messages that are humorous, ironic, or sincere; while discourse analysis treats all such utterances as meaningful social actions, future research could usefully explore the specific rhetorical effects of humor in normalizing drinking. Some user-generated content also featured in recurring discussion threads on palm wine, bitters, and alcohol’s perceived health benefits, but the study did not conduct a detailed interactional analysis of conversational sequences. Future studies could examine how alcohol-related meanings are negotiated, challenged, or reinforced across extended comment exchanges. The dataset is limited to content from Facebook and TikTok. Given that social media participation is shaped by access, digital literacy, and platform preferences, the data may overrepresent more digitally active populations and exclude interactions occurring in closed or less visible digital spaces. The study’s focus on Ghana limits its generalizability, though the hybridity observed (global platforms combined with local cultural forms) may be relevant to other African social media contexts. Finally, the absence of user demographic data precludes analysis of how gender, age, and social status shape these discourses. The few messages referencing women (eg, P63) suggest gendered patterns that warrant further investigation. Consistent with a discourse analytic approach, the study does not seek to measure the prevalence of attitudes or behaviors, but to examine how meanings are constructed and circulated. The findings should therefore be understood as illustrative of dominant and recurring discursive patterns, not as representative of the Ghanaian population as a whole.

### Conclusions

This study provides one of the first in-depth, discourse-analytic accounts of how alcohol is represented as a resource for emotional coping and social interaction in Ghanaian social media. It demonstrates that users draw on a set of interpretative repertoires—alcohol as emotional repair, as a social connector, and as a routine mood regulator—to construct drinking as a reasonable, effective, and culturally endorsed response to the demands of everyday life. These repertoires are further reinforced by a commercial-lay hybrid discourse that links alcohol products to health, vitality, and emotional well-being, while adapting broader narratives about drinking for self-care and leisure to Ghanaian expressive contexts. The findings show that alcohol-related discourse cannot be understood only through risk or harm. The meanings attached to drinking are also shaped by emotional needs, social relationships, cultural practices, and everyday understandings of well-being. These constructions may compete with risk-based health messages when such messages do not engage with the meanings that make alcohol appealing or useful in everyday life. Health communication interventions could therefore be tailored to address constructions of alcohol as emotional relief, social connection, or everyday mood regulation, while offering practical alternatives that acknowledge the emotional and social functions attributed to drinking. The study highlights the value of examining alcohol use as a discursive and culturally situated practice, particularly within participatory digital spaces where commercial and everyday forms of alcohol-related communication increasingly intersect.

## Abbreviations

GhPE: Ghanaian Pidgin English
WHO: World Health Organization
